# Development of mental health first aid guidelines for Aboriginal and Torres Strait Islander people experiencing problems with substance use: a Delphi study

**DOI:** 10.1186/1471-244X-10-78

**Published:** 2010-10-08

**Authors:** Laura M Hart, Sarah J Bourchier, Anthony F Jorm, Leonard G Kanowski, Anna H Kingston, Donna Stanley, Dan I Lubman

**Affiliations:** 1 Orygen Youth Health Research Centre, University of Melbourne, Parkville, Victoria, Australia; 2 Aboriginal Mental Health and Drug & Alcohol, Greater Western Area Health Service, New South Wales Department of Health, Orange, New South Wales, Australia; 3 Turning Point Alcohol and Drug Centre, Eastern Health and Monash University, Fitzroy, Victoria, Australia

## Abstract

**Background:**

Problems with substance use are common in some Aboriginal communities. Although problems with substance use are associated with significant mortality and morbidity, many people who experience them do not seek help. Training in mental health first aid has been shown to be effective in increasing knowledge of symptoms and behaviours associated with seeking help. The current study aimed to develop culturally appropriate guidelines for providing mental health first aid to an Aboriginal or Torres Strait Islander person who is experiencing problem drinking or problem drug use (e.g. abuse or dependence).

**Methods:**

Twenty-eight Aboriginal health experts participated in two independent Delphi studies (n = 22 problem drinking study, n = 21 problem drug use; 15 participated in both). Panellists were presented with statements about possible first aid actions via online questionnaires and were encouraged to suggest additional actions not covered by the content. Statements were accepted for inclusion in the guidelines if they were endorsed by ≥ 90% of panellists as either 'Essential' or 'Important'. At the end of the two Delphi studies, participants were asked to give feedback on the value of the project and their participation experience.

**Results:**

From a total of 735 statements presented over two studies, 429 were endorsed (223 problem drinking, 206 problem drug use). Statements were grouped into sections based on common themes (n = 7 problem drinking, n = 8 problem drug use), then written into guideline documents. Participants evaluated the Delphi method employed, and the guidelines developed, as useful and appropriate for Aboriginal and Torres Strait Islander people.

**Conclusions:**

Aboriginal health experts were able to reach consensus about culturally appropriate first aid for problems with substance use. Many first aid actions endorsed in the current studies were not endorsed in previous international Delphi studies, conducted on problem drinking and problem drug use in non-Indigenous people, highlighting the need for culturally specific first aid strategies to be employed when assisting Aboriginal or Torres Strait Islander people.

## Background

Australia's diverse groups of Aboriginal and Torres Strait Islander peoples constitute 2.3% of the population [1]. The most recent National Drug Strategy Household Survey reported that rates of illicit drug and alcohol use are significantly higher in this population than in the non-Aboriginal population. Use of illicit drugs in the twelve months prior to survey was reported by 24.2% of Aboriginal people, compared to 13.0% of the general population [2]. In addition, the survey found that while Aboriginal people are more likely to abstain from drinking than the general Australian population (23.4% versus 16.8%), those who choose to drink are more likely to consume alcohol at risky or high-risk levels, compared to the general population (27.4% versus 20.1%) [2]. Other research has found similar patterns, and in some Aboriginal communities rates of alcohol, cannabis and inhalant use are all reported to be higher than in the general population [3-8].

Elevated levels of substance use and abuse are of concern because they are associated with substantial, yet preventable mortality, morbidity and social burden [2,3,9]. For example, young Aboriginal people (aged 18-34 years) who have recently used illicit drugs are less likely to report being in excellent or very good health (41% compared with 58%) [4]. In 2003, alcohol was the fifth leading cause of disease burden and injury among Aboriginal and Torres Strait Islander Australians, responsible for 6.2% of the total disease burden and 7% of all deaths [9,10]. Furthermore, Aboriginal females were 7.9 times more likely to experience disease, injury or death related to harmful alcohol use or alcohol dependence, than their non-Aboriginal counterparts [9]. In addition, alcohol and drug use is linked to elevated levels of mental health problems, family violence, contact with the criminal justice system and suicide in Aboriginal communities [9,11-14].

Despite the significant impact substance abuse has on individuals and communities, many people who experience problems with drinking or drug use do not seek help. Indeed, compared with other mental illnesses, Australians with substance use disorders are the least likely to seek help for their problem, with only one quarter of people who meet criteria for a substance use disorder seeking help within the previous 12 months [15]. While these data do not specify the rates of service use among Aboriginal Australians, other data sources illustrate that Aboriginal and Torres Strait Islander people are more likely to delay contact with services until a problem becomes acute and is therefore more severe and difficult to treat [3]. Failure or delay in seeking help increases the risk of associated harms, such as the development of comorbid physical, mental health and social and emotional wellbeing problems [16].

The social networks of people with drinking or drug use problems are known to have an important impact on an individual's decision to seek treatment or to stop using [17]. In Aboriginal communities in particular, broad family connections are central to identities and livelihoods. Here, the social network involves increased responsibility and reciprocity, which is both greatly affected by substance abuse, but also offers great influence for change [14]. However, community members often lack the knowledge and skills in how to recognise problems and to assist a person in seeking out professional help [18,19]. Interventions that develop better recognition of symptoms and strategies for effective help seeking are therefore needed, particularly in Aboriginal communities that are struggling with elevated levels of substance use.

One intervention that has been shown to be effective in improving mental health literacy (the knowledge and beliefs about mental illnesses and substance use disorders that aid their recognition, management or prevention) [19,20], is the training provided by the Mental Health First Aid Training and Research Program (MHFA). Mental health first aid is defined as *the help provided to a person developing a mental health problem or in a mental health crisis. The first aid is given until appropriate treatment is received or the crisis resolves *[21]. Here, the term 'mental health problem' refers to any behavioural or psychiatric disturbance which negatively affects a person's mental health. It includes both diagnosable mental illnesses and substance use disorders, as well as symptoms of these disorders which do not meet a clinical threshold, yet cause a person distress or disability. The term 'mental health crisis' refers to a state in which someone is either very distressed or very unwell and there is an increased risk of harm. Examples include drug-induced psychosis and withdrawal states.

Mental health first aid techniques are taught in a 12 hour training course, provided by MHFA, which was established in 2001 in response to poor mental health literacy in the community [22]. This course includes information on how to assist someone with a substance use problem and someone in a substance use crisis (e.g., psychosis associated with intoxication). Several trials have been conducted to evaluate the effects of the MHFA program and these have found it to be effective in: increasing recognition of mental illness, changing beliefs about treatment to be more like those of health professionals, reducing stigmatizing attitudes, increasing confidence in providing help to someone with a mental illness, increasing the amount of help provided to others and improving the mental health of participants [22].

The Aboriginal and Torres Strait Islander Mental Health First Aid program (AMHFA), a cultural adaptation of the MHFA course, began in 2007. The AMHFA course differs from the general course in that it recognises the historical, cultural and political forces that have affected Aboriginal mental health in Australia, and in recognising the unique barriers Aboriginal and Torres Strait Islander people face in seeking out appropriate mental health care. The 14-hour course is delivered by trained Aboriginal and Torres Strait people to predominantly Aboriginal audiences [23]. The AMHFA program has undergone an initial evaluation with results demonstrating that the course is culturally appropriate, empowering for Indigenous people and provides information that is highly relevant and important in assisting Aboriginal people with a mental illness [23].

In order to ensure that the mental health first aid techniques taught to the public in these courses are as evidence based as possible, research has been carried out to develop guidelines on what constitutes best practice first aid. To date, guidelines have been developed for providing first aid in a range of mental health related crises and for a range of developing mental illnesses [24-31]. Separate guidelines for providing culturally competent mental health first aid to Aboriginal and Torres Strait Islander people have also been developed. These include guidelines for assisting in the case of depression, psychosis, suicidal thoughts and behaviours, deliberate self injury and trauma and loss [32]. A sixth guideline titled *Cultural Considerations and Communication Techniques *was also developed to promote the importance of understanding and respecting Aboriginal and Torres Strait Islander culture while providing mental health first aid [32].

The purpose of the current research was to develop culturally appropriate guidelines for providing mental health first aid to an Aboriginal or Torres Strait Islander person who is experiencing problem drinking or problem drug use. The guidelines are intended to increase mental health literacy and improve the capacity of Aboriginal communities to intervene early and seek appropriate help for problems with substance use.

## Methods

Detailed information about how the Delphi method is employed to develop culturally appropriate mental health first aid guidelines has been described elsewhere [32]. As the current research followed the same process as that previously described, only essential detail and variations are described here. Two Delphi studies were completed; one on problem drinking and one on problem drug use. While the studies were completed independently, both followed the same procedure, except where specified below.

### Participants

Participants were required to meet three inclusion criteria: to identify as an Aboriginal or Torres Strait Islander person; to be currently working in or to have had previous experience in the fields of mental health or substance use treatment; and to have an excellent knowledge of Aboriginal substance use and the issues involved when Aboriginal people seek assistance for problems with substance use. Eligible participants were identified through previous research participation [32] and through the register of accredited Aboriginal and Torres Strait Islander Mental Health First Aid Instructors, maintained by the MHFA organisation. To become an Instructor, an Aboriginal or Torres Strait Islander person must have a high level of mental health knowledge and currently be working for an organisation that supports the improvement of mental health literacy in Aboriginal communities [23].

While there is no perfect sample size for conducting a Delphi study, the current research aimed to have 30 panel members for each study, in order to balance issues encountered with a large sample size (of 60 or more), where consensus is difficult to reach, with that of a small sample (of 15 or less), where views of particular individuals can strongly influence study results [33,34]. Informed consent was implied by responding to online questionnaires. This research was granted human research ethics committee approval by the University of Melbourne. Participants were paid $A150 for each survey round completed.

### Instruments

The Delphi method involves presenting information to experts for rating. Where information just fails to reach consensus, iterations are completed until consensus is achieved. The current study required participants to rate statements describing possible mental health first aid actions, on a five-point scale of importance, which included the options: 'Essential', 'Important', 'Don't know/depends', 'Unimportant' and 'Should not be included'. Participants responded via online questionnaires hosted by surveymonkey software http://www.surveymonkey.com.

The first round statements were constructed from recommendations uncovered in systematic literature searches. Websites, online forums, information brochures, leaflets or hand-outs from service providers or information centres, medical journals and online databases, were all searched for any information about how to assist an Aboriginal person experiencing problem drinking or problem drug use. Terms used in the problem drinking study included (grog OR alcohol OR drinking OR booze) AND (Aboriginal OR Indigenous). Terms used in the problem drug use study included (Aboriginal OR Indigenous) AND (drug OR substance OR inhalant) AND (use OR misuse OR problem OR addiction) AND (help OR first aid OR early intervention). Any links appearing on websites, or references in journal articles, which the authors thought may contain useful information, were also followed.

In addition to the statements developed from the searches, statements that were developed in two international Delphi studies on problem drinking and problem drug use [28,31] were also incorporated into the first round questionnaires. This was done to ensure that any gaps in the Aboriginal-specific literature were still considered by the panel. Each questionnaire was broken into separate sections based on common themes in statements. In the problem drinking study, statements were grouped in 7 sections (see Table 1), and in the problem drug use study, into 8 (see Table 2).

**Table 1:** Statement Themes - Problem Drinking study

**Section 1. Problem drinking**
1.1 What the first aider needs to know about problem drinking
1.2 Understanding problem drinking in the community
1.3 Knowing when the person needs help for their drinking
**Section 2. Talking to the person about their problem drinking**
2.1 Discussing the problem
2.2 Under standing the person's reaction
2.3 Providing information about problem drinking
2.4 Encouraging the person to change
**Section 3. If the person wants to change**
3.1 Initiating change
3.2 Dealing with the social pressure to drink
3.3 Encouraging other supports
**Section 4. Seeking professional help**
4.1 Professional help seeking
4.2 Discussing professional help with the person who wants to change
**Section 5. If the person does not want help**
5.1 If the person is unwilling to change their drinking behaviour
5.2 If the person is unwilling to seek professional help
**Section 6. Intoxication**
6.1 What the first aider needs to know about intoxication
6.2 If the person is intoxicated
6.3 Talking to the intoxicated person
6.4 Getting the intoxicated person home or to a safe place
6.5 What to do if the intoxicated person becomes aggressive
**Section 7. Withdrawal**

**Table 2:** Statement Themes - Problem Drug Use study

**Section 1. Problem drug use**
1.1 What the first aider needs to know about problem drug use
1.2 How to recognise problem drug use
**Section 2. Approaching the person about their problem drug use**
2.1 Preparing to approach the person
2.2 General principles for talking to the person
2.3 When to talk to the person
2.4 What to say to the person
2.5 If the person is pregnant or breastfeeding
2.6 If the person is caring for a child
**Section 3. Information and support for the person who wants to stop using drugs**
3.1 Self help
3.2 Helpful information
3.3 Support
3.4 Helping the person deal with social pressure to take drugs
3.5 Harm reduction
3.6 Laws around drug use/possession
**Section 4. When to disclose the person's drug use**
**Section 5. If the person is unwilling to change**
**Section 6. Encouraging the person to seek professional help**
6.1 Suggesting help
6.2 Types of help
6.3 Making the appointment
**Section 7. If the person is unwilling to seek help**
**Section 8. Drug affected states**
8.1 Understanding drug affected states
8.2 Sniffing
8.3 Responding to medical emergencies
8.4 If the person becomes agitated or aggressive
8.5 What to do if the first aider cannot de-escalate the situation

### Procedure

Once all participants had rated the first aid action statements, responses were analysed by obtaining percentage endorsement scores for each statement. Statements were then placed into one of three categories.

1. If between 90-100% of panel members rated a statement as either 'Essential' or 'Important', the statement was endorsed as a guideline.

2. If between 80-89% of panel members endorsed the statement as 'Essential' or 'Important', then the statement was entered into a second questionnaire to be re-rated.

3. If neither of the above conditions were met, then the statement was excluded from the guidelines.

At the end of the first round questionnaires, panel members were encouraged to provide feedback on any first aid strategies not yet covered. New statements were developed from this feedback and presented in a second round, along with statements from the first round that required re-rating. The same criteria for endorsing, excluding and re-rating statements were applied in the second rounds, with one exception. If a statement was re-rated and again failed to achieve a consensus of between 90 and 100 percent across the panel, it was then excluded. Only those statements that had been entered as new statements in the second round, and afterward fell into the re-rate category, were entered into a third round questionnaire. In total, three rounds of questionnaires were developed for the problem drinking study and two rounds for the problem drug use study (a total of 5 questionnaires).

All statements that were endorsed as either 'Essential' or 'Important' by ≥ 90% of panel members were then written into a guideline document. Two authors (SJB and LMH) drafted the guidelines by writing the list of endorsed statements into sections of prose based on common themes. A number of drafting iterations, overseen by a working group (AFJ, LGK, DS, DIL), were completed before the final document was produced and a copy was sent to each panel member for review. The guidelines are available for free download from the MHFA website http://www.mhfa.com.au/Guidelines.shtml.

### Evaluation

To assess the panel members' satisfaction with the research method and developed guidelines, participants were invited to complete an online feedback questionnaire at the end of the two Delphi studies. Respondents were encouraged to comment on the appropriateness of the contact methods, research methods, language and concepts used throughout the studies. They were also asked how culturally appropriate and useful they thought the developed guidelines would be to Aboriginal people in the future. The feedback survey contained 14 statements that described the research experience (e.g. *I thought participating in this research was worthwhile*). Participants were asked to respond by selecting where their opinion fell on a 5-point scale of agreement; 'Strongly Agree', 'Agree', 'Neither Agree nor Disagree', 'Disagree', and 'Strongly Disagree'.

## Results

### Participants

Twenty-eight panel members were recruited across the two studies (13 female, 15 male, age range = 28-59 years). Twenty-two panel members participated in the problem drinking study and 21 in problem drug use. Of the 22 participants recruited for the problem drinking study, 15 also participated in the problem drug use study. Table 3 outlines how many panel members responded to each round of the two studies. There was a high retention rate across rounds of questionnaires and across the two studies (86% across rounds for problem drinking, 100% across rounds for problem drug use and 72% from study 1 to study 2).

**Table 3:** Number of respondents per round for each questionnaire topic

	Problem drinking	Problem drug use
Round 1	21	21
Round 2	19	21
Round 3	19	n/a*

* Only two rounds were completed in the problem drug use study as none of the first aid action statements, which were rated for the first time in round 2, fell into the re-rate category prompting a third and final round.

Participants were recruited from across Australia including: Australian Capital Territory (n = 4), New South Wales (n = 8), Northern Territory (n = 1), Queensland (n = 8), South Australia (n = 3), Victoria (n = 2) and Western Australia (n = 2). Tasmania was the only state without representation on the panel. Having a geographical spread of panel members was thought to be important for the representation of different experiences and attitudes of Aboriginal communities across Australia. It is also important to note that only 2 participants identified as Torres Strait Islander or both Aboriginal and Torres Strait Islander. The remaining 26 participants identified as Aboriginal.

Participants were employed in a range of different health services, including alcohol and drug services, Aboriginal medical services, universities, government health services, social services, cultural resource centres and counselling services, prisons and forensic services. Panel members experience in the mental health field was extensive (5 years or less = 10.5%, 6-10 years = 42.1%, 11-15 years = 21.1%, 16-20 years = 10.5%, 21 years or more = 15.8%). While no data is available to quantify participants' specific experiences of working within alcohol and drug services, all participants worked in positions that involved contact with or treatment of Indigenous people with substance use problems. Approximately one third of panel members had obtained a post-graduate qualification (Diploma = 21.1%, Bachelor Degree = 42.1%, Graduate Diploma = 15.8%, Masters degree 21%).

### First aid actions

#### Endorsed statements

Of the 735 statements presented to participants over the two studies, 429 were endorsed as either 'Essential' or 'Important' to the development of guidelines for providing mental health first aid to an Aboriginal or Torres Strait Islander person. A list of all endorsed statements can be found in Additional File 1: Endorsed Statements Problem Drinking and Additional File 2: Endorsed Statements Problem Drug Use. Table 4 lists the number of statements presented in each Delphi study.

**Table 4:** Number of statements presented, endorsed and rejected in each Delphi study

		Problem drinking	Problem drug use
Round 1	New statements	313	316
	Statements being re-rated	0	0
	Total no. of statements	313	316
	Statements endorsed	192	177

Round 2	New statements	13	3
	Statements being re-rated	38	49
	Total no. of statements	51	52
	Statements endorsed	30	29

Round 3	New statements	0	0
	Statements being re-rated	3	0
	Total no. of statements	3	0
	Statements endorsed	1	0

Total statements		325	319
**Total endorsed statements**		**223**	**206**
Total rejected statements		144	162

#### Rejected statements

Some statements were strongly rejected by the panel, with a majority of participants rating a statement as either 'Unimportant' or 'Should not be included' (see Additional File 3: Strongly Rejected Statements). Across the 2 Delphi studies 11 items were rejected with strong consensus (50% or more of panel members rated an item as either 'Unimportant' or 'Should not be included'). Both studies had a similar number of strongly rejected statements, all of which were rejected in the first round.

Other statements were rejected because there was a lack of consensus within the panel. For instance, some statements failed to be endorsed because even after a second rating, the statement just failed to achieve 90% consensus. In both studies, the majority of the rejected statements came from the section on how to assist when the person is intoxicated.

#### Re-rated statements

In the problem drinking study, 41 statements were neither rated highly enough to be endorsed or weakly enough to be rejected, so were resubmitted to the panel in the next round. In the problem drug use study, 49 statements were re-rated in the second round, however, there were no statements that were entered for the first time in round 2 and afterwards fell into the re-rate category. As such, there was no third round.

### Evaluation

Nineteen of a possible 28 participants responded to the feedback survey (68%). Table 5 shows responses to statements included in the survey. Of particular interest were the responses to statements that were designed to assess the cultural appropriateness, the utility and perceived quality of the guidelines produced. For instance, 94.7% of the panel responded with either 'Strongly Agree' or 'Agree' to the statement *I thought the guidelines were culturally appropriate*; 89.5% to the statement *I would recommend the guidelines to other people*; and 100% to the statement *I believe the guidelines will benefit Aboriginal people*.

**Table 5:** Statements from the panel member feedback survey

Feedback statement	Strongly agree	Agree	Neither	Disagree	Strongly disagree
I thought the guidelines were easy to follow.	52.6	47.4	0	0	0
I thought the guidelines were too long.	0	10.5	36.8	52.6	0
I thought the guidelines used appropriate language.	26.3	63.2	10.5	0	0
I thought the language used in the guidelines was too clinical.	0	5.3	21.1	73.7	0
I thought the guidelines covered the appropriate issues.	36.8	52.6	5.3	0	0
I thought the guidelines were culturally appropriate.	36.8	57.9	5.3	0	0
I believe the guidelines will benefit Aboriginal people.	63.2	36.8	0	0	0
I would recommend the guidelines to other people.	63.2	26.3	0	10.5	0
I thought the time commitment was appropriate.	42.1	47.4	0	10.5	0
I thought the remuneration was appropriate.	42.1	52.6	5.3	0	0
I thought participating in this research was worthwhile.	89.5	5.3	0	5.3	0
I enjoyed participating in the Delphi research.	68.4	21.1	0	5.3	0
I believe the Delphi process can be of benefit to Aboriginal people.	73.7	21.1	5.3	0	0
I would recommend the Delphi method for other research projects for Aboriginal people.	73.7	21.1	0	0	5.3

Statements regarding the appropriateness of the Delphi research method also received a high level of agreement, with 94.7% of participants responding with either 'Strongly Agree' or 'Agree' to the statements *I believe the Delphi process can be of benefit to Aboriginal people *and *I would recommend the Delphi method for other research projects for Aboriginal people*.

## Discussion

By engaging Aboriginal health workers with expertise in the areas of substance use and mental health, this research aimed to develop culturally appropriate guidelines for providing mental health first aid to an Aboriginal or Torres Strait Islander person experiencing problem drinking or problem drug use. Despite geographical, cultural and professional differences, panel members were able to reach consensus on a range of first aid techniques, from understanding the stages of change and discussing drinking or drug use problems, to encouraging professional help and providing assistance in a medical emergency.

Sixty-nine percent of the first aid statements in the problem drinking study, and 65% of the statements in the problem drug use study, were endorsed by the panel. This compares to 52% and 46% of the problem drinking and problem drug use international Delphi studies respectively [28,31]. While the rate of endorsement is higher in the current studies, this appears to be an artefact of having an entirely professional sample, with no consumer or carer panels, rather than a willingness to endorse more strategies. This is exemplified by the fact that many statements about encouraging professional help and providing information on problem use failed to be included in the international problem drinking guidelines, not because they were rejected by panel members, but because the different panels failed to reach a consensus on their level of importance. For example, in the international problem drug use study, the statement *The first aider should encourage the person to seek professional help *was rejected because it failed to reach a high enough level of endorsement from the consumer and clinician panels (carers 77.4%, consumers 44.8% and clinicians 59.3%). From examination of the level of endorsement given by each panel, it appears that the autonomy of the consumer clashed with the desire of the carers to advocate for professional help on the person's behalf [28]. In contrast, a number of statements about encouraging professional help were endorsed in the current study on problem drug use: (1) *The first aider should encourage the person to seek appropriate professional help as soon as possible; *(2) *The first aider should ask the person if they would like to get professional help; *(3) *The first aider should encourage the person to seek professional help; *(4) *The first aider should discuss with the person why they need professional help*. The endorsement of these statements appears to show that when there are not different perspectives and values between panels, a much more direct line of advocacy has appeared when it comes to the first aider suggesting someone seek professional help.

The lack of consumer and carer perspective is acknowledged as a limitation of the current research. It would have been beneficial to the development of the guidelines to include the unique perspective of those with the lived experience. As consumers and carers are the individuals who are most likely to receive mental health first aid, or to provide it, they have a valuable knowledge base that is not necessarily represented in clinical or professional expertise. However, finding a sufficient sample of Aboriginal or Torres Strait Islander people who had experienced a past drinking or drug use problem, or cared for someone who did, and furthermore were comfortable reflecting on their experience in the public domain, proved impractical.

While the majority of statements endorsed in the current Delphi study and the statements endorsed in the previous international studies overlapped, there were also points of difference. In particular, the current study included four novel themes not seen in the previous studies: information about calling the police as a last resort when trying to de-escalate aggressive behaviours, the importance of understanding the social environment and its impact on substance use, and the need for specific harm reduction strategies for Aboriginal and Torres Strait Islander people.

### Police involvement in de-escalating aggressive behaviours

In each of the current studies, two statements were endorsed that mention the need to contact police while assisting someone who is intoxicated. In the problem drinking study the following statements were endorsed: *The first aider should be aware that if the person needs to be contained, sobering up shelters and drug and alcohol resource centres are preferable to police lock-ups, because they can help the person stay safe, learn about their drinking and its risks, and get some professional help*; and *If the person becomes aggressive, the first aider should only call the police if all other avenues of de-escalation have been exhausted*. The former statement was also endorsed in the problem drug use study, along with the statement *The first aider should know that the police will only be called to an emergency if the ambulance officers feel they can't control what is happening. The emergency workers first priority is to save the life of the person who is unwell*. While the international Delphi studies endorsed statements about police involvement, the Aboriginal and Torres Strait Islander experts appeared to be more reluctant; only items that specifically focused on police as a last resort were endorsed in the current studies. The authors suspect that the findings of the 1988 Royal Commission into Aboriginal Deaths in Custody may in some part explain the need for the first aider to take particular care in avoiding police custody for an Aboriginal person who is intoxicated. The commission found that Aboriginal people were "grossly over-represented in apprehensions for public drunkenness" and that while intoxication is not only a factor leading to people being in custody, it is also, and more importantly, a factor in "increasing their vulnerability to death in custody" [35].

### The social environment and its impact on substance use

Drinking and drug use behaviours are strongly influenced by the social and cultural environment in which they take place [36-38]. In the current problem drinking study, a number of statements were endorsed that recognise the role of the community or group on individual behaviour. Nine separate statements were endorsed, which refer to the need for the first aider to consider and draw upon the role of the community in the person's substance use (see Items 28 - 36, 54 in Additional file 1). One example is the statement: *If drinking problems in the person's community are widespread, the first aider should speak to community leaders about initiating change*. This theme was not as strongly apparent in the problem drug use study, with only four items endorsed relating to the role of community influencing the person's drug use (see items 2, 21, 87, 94). By comparison, the international guidelines include very little reference to the impact a person's social environment can have on their use. The inclusion of statements that reflect the importance of the social environment in the current studies may reflect broader differences between Indigenous and non-Indigenous cultures in Australia. Australian Aboriginal culture has long been recognised as collectivistic rather than individualistic, because in Aboriginal communities the rights and responsibilities of the group tend to be placed above the rights and responsibilities of the individual [39-41]. The need to address problem drinking and drug use by using collective action, rather than individual intervention, may therefore be an appropriate first aid strategy when assisting an Aboriginal or Torres Strait Islander person within their community.

### Specific harm reduction strategies

Engaging the use of a sobering-up centre, a night patrol, or respected Elder, were all novel first aid techniques that were gleaned from the literature search on assisting an Aboriginal person with problem drinking or problem drug use. The importance of recognising possible environmental harms, in places where Aboriginal people are more likely to drink or take drugs, were also novel inclusions. For example in the problem drug use study the statement *The first aider should provide the person with information about harm reduction strategies specifically for Aboriginal and Torres Strait Islander people *was endorsed. The specific strategies that were written into the final guideline document include: *Not using drugs near lakes, rivers or the sea where the person could drown *and *not using drugs near busy roads where they could be run over*. These reflect the fact that some Aboriginal people are more likely to drink alcohol in public places and are consequently at an increased risk of specific environmental harms [40,41]. In addition, information about the harms associated with sniffing inhalants (e.g. glue, paint or petrol) was also endorsed by the expert panel members, as it is recognised that some Aboriginal communities struggle with sniffing behaviour, particularly among their young men [6,17,42].

### Evaluation

In Australia, the National Health and Medical Research Council has guidelines for Ethical Conduct in Aboriginal and Torres Strait Islander Health Research. According to this document, a central tenet of ethical research with Aboriginal and Torres Strait Islander people is reciprocity, or the need to ensure that "research outcomes include equitable benefits of value to Aboriginal and Torres Strait Islander communities or individuals" [43]. In order to establish that the current Delphi studies had employed culturally appropriate methods and developed resources that will be of benefit to Australia's Indigenous people, the current research sought feedback from its panel members. Consistent with the findings of a previous Delphi study evaluation [32], the current research received a high level of endorsement from its participants as a culturally appropriate method. Furthermore, the guidelines developed by this research were considered to be of benefit to Aboriginal people. While this is encouraging, it must be noted that 72% of the panel members in the current study had previously participated in similar Delphi research, so the sample may have been self-selected to be favourable to this type of research [32].

### Future directions

The developed guidelines will be used to update the existing AMHFA course and will be taught in training programs across Australia. In addition Australia's *beyondblue: the national depression initiative *has developed a dissemination program whereby copies of the guidelines are made available free of charge to community members. This resource is expected to be particularly valuable to health, education and community resource centres across Australia who engage Aboriginal and Torres Strait Islander clients.

Further research and evaluation, however, is still needed in order to understand the impact the guidelines ultimately have on increasing mental health literacy and help seeking for problem drinking or drug use.

## Conclusions

In the current study, a number of important themes emerged from the endorsed first aid action statements. A number of these themes were novel and were not present in the international Delphi studies on problem drinking and problem drug use, which reiterates the importance of developing culturally specific mental health first aid resources for Indigenous people. In particular, when assisting an Aboriginal or Torres Strait Islander person with problem drinking or problem drug use, a first aider should take care to understand the role of social environment on the person's use, should provide culturally specific information about harm-reduction strategies, and in the event that the person they are assisting is intoxicated, take care not to involve the police unless necessary. Evaluations of the Delphi method suggested that it is a research method that is considered appropriate and useful for Aboriginal and Torres Strait Islander people in Australia.

## Competing interests

A number of authors have an affiliation with the Mental Health First Aid Training and Research Program. AFJ is the scientific director, LGK is the co-ordinator of the Aboriginal Mental Health First Aid Program and LMH is a research assistant for the Aboriginal Mental Health First Aid Program. The publication of this manuscript may benefit the Mental Health First Aid Training and Research Program by advertising the concept of mental health first aid for Aboriginal Australians.

## Authors' contributions

For the problem drinking study: LMH carried out the systematic literature search, was involved in panel member recruitment, drafted the surveys, carried out the data collection and analysis, chaired the working group which discussed and modified the survey and guideline drafts, drafted the guidelines, and drafted the manuscript. For the problem drug use study: SJB carried out the systematic literature search, was involved in panel member recruitment, drafted the surveys, carried out the data collection and analysis, chaired the working group which discussed and modified the survey and guideline drafts, drafted the guidelines, and assisted with drafting of the manuscript. For both studies: AFJ participated in the conception and design of the Delphi research protocol, acted as the chief investigator, participated in the working group and helped with the drafting of the manuscript. LGK was involved in design and co-ordination of the study, assisted with panel member recruitment and participated in the working group. DS participated in the working group and provided expert cultural consultation on the guideline and manuscript drafts. AHK contributed to the development of the first round questionnaires. DIL participated in the working group and provided expert consultation on substance related issues. All authors read and approved the final manuscript.

## Pre-publication history

The pre-publication history for this paper can be accessed here:

http://www.biomedcentral.com/1471-244X/10/78/prepub

## Supplementary Material

Additional file 1 **Endorsed Statements Problem Drinking**. Endorsed first aid action statements from the problem drinking study. Click here for file

Additional file 2 **Endorsed Statements Problem Drug Use**. Endorsed first aid action statements from the problem drug use study. Click here for file

Additional file 3 **Strongly Rejected Statements**. First aid action statements from both the problem drinking and problem drug use studies. Click here for file
