# Supplementary material for: Development of mental health first aid guidelines for Aboriginal and Torres Strait Islander people experiencing problems with substance use: a Delphi study
Source: BMC Psychiatry. 2010 Oct 8;10:78. doi: 10.1186/1471-244X-10-78 (PMC2964528; doi:10.1186/1471-244X-10-78)
Supplement: Additional file 1 — Endorsed Statements Problem Drinking. Endorsed first aid action statements from the problem drinking study. [file 1471-244X-10-78-S1.DOC]

Endorsed statements from the Delphi study “First Aid for Problem Drinking”

| **Statement number** | **Round endorsed** | **Endorsed statements** |
| --- | --- | --- |
| ***Section 1. Problem drinking***  *1.1 What the first aider needs to know about problem drinking* | | |
| 1 | 1 | The first aider should be aware that the effects of alcohol vary from person to person depending on how fast they drink, whether they have eaten anything, whether they are male or female, how big they are, how old they are and whether they are used to drinking or not. |
| 2 | 1 | The first aider should be aware that some people will drink and use other drugs (such as cannabis or amphetamines) at the same time. |
| 3 | 1 | The first aider should be aware that drinking alcohol is linked to increased episodes of incarceration, violence (in both family and community), risky behaviour (such as drink driving), accidents & injuries, hospitalisation, ill-health, suicide and homicide. |
| 4 | 1 | The first aider should be aware that, according to research, Aboriginal people who choose to drink are more likely to drink at risky or high-risk levels than to drink at low-risk levels. |
| 5 | 1 | The first aider should be aware that there are many good and freely available resources for Aboriginal people who are experiencing problem drinking. |
| 6 | 1 | To learn more about problem drinking, the first aider should find some information specifically for Aboriginal people. For instance, from the local Aboriginal or community health service, drug and alcohol service, doctor's clinic, in the library or on the internet. |
| 7 | 1 | The first aider should be aware that they can speak to professionals, such as a drug and alcohol or community health workers or doctors, to get information about how to approach or help the person with their problem drinking. |
| 8 | 1 | The first aider should be able to recognise the symptoms of alcohol dependence. |
| 9 | 1 | The first aider should be able to recognise the symptoms of alcohol abuse. |
| 10 | 1 | The first aider should be able to recognise the symptoms of alcohol poisoning (overdose). |
| 11 | 1 | The first aider should be able to recognise some of the early warning signs for problem drinking. |
| 12 | 1 | The first aider should know some tips for low-risk drinking. |
| 13 | 1 | The first aider should have general knowledge of some of the reasons why people drink alcohol to excess. |
| 14 | 1 | The first aider should know the short and long-term consequences of risky and high-risk drinking. These may include physical, mental or social problems. |
| 15 | 1 | The first aider should know that mental health problems can be caused by, or made worse by, drinking. |
| 16 | 1 | The first aider should be aware that drinking is often used to cope with underlying emotional distress or mental illness. This is often called “self-medication”. |
| 17 | 1 | The first aider should be aware that if the person has underlying emotional distress or mental health issues these may need to be addressed in order for a person to stop their problem drinking. |
| 18 | 1 | The first aider should be aware that it is not possible to force a person to change their behaviour. |
| 19 | 1 | The first aider should be aware that the person is the only one who can make the decision to change their drinking behaviour. |
| 20 | 1 | The first aider should be aware that a person’s willpower and self-resolve is not always enough to help them stop problem drinking. |
| 21 | 1 | The first aider should be aware that it is not easy to change drinking habits. |
| 22 | 1 | The first aider should be aware that giving advice alone may not help the person change their drinking behaviour. |
| 23 | 1 | The first aider should be aware that sobering up (detoxification) is only part of recovery and many lifestyle changes are required to change drinking behaviours. |
| 24 | 1 | The first aider should be aware that if the person has been drinking heavily for a long time, stopping suddenly can be very dangerous to their health. |
| 25 | 1 | The first aider should be aware that the person may relapse once or several times before changing their drinking patterns. |
| 26 | 1 | The first aider should be aware that abstinence (stopping all grog) is not the only alternative to problem drinking. |
| 27 | 1 | The first aider should be aware that the person’s family may deny or make excuses for the person’s drinking behaviour. |
| 28 | 2 | The first aider should be aware that, according to research, the percentage of Aboriginal people who choose to drink is lower than the percentage of non-Aboriginal people who choose to drink. |
| 29 | 2 | The first aider should be aware that, according to research, Aboriginal people are more likely to be non-drinkers than are non-Aboriginal people. |
| 30 | 2 | The first aider should be aware that according to research, the percentage of Aboriginal people who experience alcohol-related disease, injury and death, is higher than the percentage of non-Aboriginal people who experience alcohol-related disease, injury and death. |
| *1.2 Understanding problem drinking in the community* | | |
| 31 | 1 | The first aider should be aware that the social pressures on the person to drink may be very strong. |
| 32 | 1 | The first aider should be aware that the person’s drinking may be part of a wider pattern of problem drinking in the community. |
| 33 | 1 | The first aider should not accept problem drinking as part of Aboriginal culture or tradition. |
| 34 | 1 | The first aider should be aware that the person's drinking behaviour can be strongly influenced by a range of factors, including who the person drinks with, their community’s attitude to drinking and where they choose to drink. |
| 35 | 1 | The first aider should be aware that the environment in which the person drinks can make it harder or easier for them to change their drinking behaviour. |
| 36 | 1 | The first aider should be aware of the attitudes that the person's community has towards drinking. |
| 37 | 1 | The first aider should be aware that drinking contributes to a lot of family violence problems. |
| 38 | 1 | The first aider should be aware that some Aboriginal communities place bans or restrictions on the sale or consumption of alcohol. |
| 39 | 1 | The first aider should be aware of what alcohol restrictions or alcohol management plans apply in the community in which they are helping. |
| 40 | 2 | The first aider should set an example by following the community's rules about alcohol. |
| 41 | 2 | The first aider should set an example by following any rules the community has about alcohol restriction. |
| *1.3 Knowing when the person needs help for their drinking* | | |
| 42 | 1 | If there are any children being affected by the person's drinking, the first aider should make the children’s safety and wellbeing a priority. |
| 43 | 1 | If the first aider becomes aware that the person’s drinking is placing the safety of others (eg. partner or family members) at risk, the first aider's priority should be to keep the people safe. |
| 44 | 1 | Warning signs for recognising problem drinking - The person is secretive about their drinking. |
| 45 | 1 | Warning signs for recognising problem drinking - The person plays down how much they drink. |
| 46 | 1 | Warning signs for recognising problem drinking - The person acknowledges they think a lot about drinking and when they’ll next get a chance to drink. |
| 47 | 1 | Warning signs for recognising problem drinking - The person is in debt because of the amount of money they spend on drinking. |
| 48 | 1 | Warning signs for recognising problem drinking - The person becomes anxious when they cannot get access to alcohol. |
| 49 | 1 | Warning signs for recognising problem drinking - The person needs to drink to help deal with certain situations. |
| 50 | 1 | Warning signs for recognising problem drinking - The person gets into arguments or has accidents because of their drinking. |
| 51 | 1 | Warning signs for recognising problem drinking - The person’s ability to perform day-to-day tasks is severely disrupted. |
| 52 | 1 | Warning signs for recognising problem drinking - The person is having marital or relationship trouble because of their drinking. |
| 53 | 1 | Warning signs for recognising problem drinking - The person has been fired, laid off or in trouble at work because of drinking. |
| 54 | 1 | Warning signs for recognising problem drinking - The person has been drink driving, charges for drink driving or other related drinking offences. |
| 55 | 1 | Warning signs for recognising problem drinking - The person is often sick or in ill health. |
| 56 | 1 | Warning signs for recognising problem drinking - The person shows increasingly irrational behaviour. |
| 57 | 1 | Warning signs for recognising problem drinking - The person suffers physically and emotionally when they have not been drinking. |
| 58 | 1 | Methods to determine if the person needs help - The first aider should describe the person’s drinking behaviour to a professional to see whether they would consider it a problem. |
| 59 | 2 | Warning signs for recognising problem drinking - The person evades questions about their drinking intake or looks uncomfortable when responding |
| 60 | 2 | Warning signs for recognising problem drinking - The person is unwilling to consider that their drinking is a problem |
| 61 | 2 | Warning signs for recognising problem drinking - The person reacts angrily when it is suggested that they have a drinking problem |
| ***Section 2. Talking to the person about their problem drinking***  *2. 1 Discussing the problem* | | |
| 62 | 1 | If drinking problems in the person’s community are widespread, the first aider should speak to community leaders about initiating change. |
| 63 | 1 | The first aider should talk with the person in a quiet, private environment. |
| 64 | 1 | The first aider should talk to the person at a time when there will be no interruptions. |
| 65 | 1 | The first aider should talk to the person when they are sober. |
| 66 | 1 | The first aider should be aware that the person may not recall events that occurred whilst they were intoxicated (i.e. they may have blacked out). |
| 67 | 1 | The first aider should talk to the person when both are in a calm frame of mind. |
| 68 | 1 | The first aider should interact with the person in a supportive way. |
| 69 | 1 | The first aider should listen carefully to the person without being judgemental. |
| 70 | 1 | The first aider should avoid the use of scare tactics. |
| 71 | 1 | The first aider should not talk in a confrontational or threatening way. |
| 72 | 1 | The first aider should not lecture the person. |
| 73 | 1 | The first aider should avoid making the person feel guilty or ashamed because of their drinking. |
| 74 | 1 | The first aider should not label the person (e.g. by calling them an addict, alcoholic or drunk). |
| 75 | 1 | The first aider should consider the person’s readiness to talk about their drinking problem by asking about areas of their life that it may be affecting, for example, their mood, work performance and relationships. |
| 76 | 1 | The first aider should allow the person to talk about their problems without interruption. |
| 77 | 1 | The first aider should talk to the person about their drinking openly and honestly. |
| 78 | 1 | The first aider should try to understand the person’s own perception of their drinking. |
| 79 | 1 | The first aider should offer to help the person and discuss what assistance they are willing to provide. |
| 80 | 1 | The first aider should be prepared to talk to the person again in the future. |
| 81 | 2 | The first aider should not force the person to admit they have a drinking problem. |
| 82 | 2 | The first aider should encourage the person to talk about any problems in their life that may be contributing to their drinking. |
| 83 | 2 | The first aider should identify and discuss the person’s behaviour rather than criticise their character, for example, “Your drinking seems to be getting in the way of your friendships” rather than “You're a pathetic drunk”. |
| 84 | 2 | The first aider should reassure the person that they are not alone and that many other people also have problems with drinking. |
| *2.2 Under standing the person’s reaction* | | |
| 85 | 1 | The first aider should not expect a change in the person’s thinking or behaviour right away; this conversation might be the first time the person has thought about their drinking as a problem. |
| 86 | 1 | The first aider should be aware that the person might not believe, or might deny, that they have a drinking problem. |
| 87 | 1 | The first aider should be aware that the person may underestimate the amount that they drink. |
| 88 | 1 | The first aider should be aware that the person may avoid questions about their drinking habits. |
| 89 | 1 | The first aider should be aware that that the person may resist the first aider’s help for a number of reasons. For instance, the person might feel that the first aider is trying to take away the person’s “right to drink”. |
| 90 | 1 | The first aider should be aware that the person may give excuses, get angry or try to blame the first aider or other family members for their drinking problem. |
| *2.3 Providing information about problem drinking* | | |
| 91 | 1 | The first aider should communicate a sense of hope and belief that the person can change their problem drinking. |
| 92 | 1 | The first aider should discuss with the person some risks associated with problem drinking. |
| 93 | 1 | If the person is pregnant or breastfeeding, the first aider should stress the dangers of drinking for the child. |
| 94 | 1 | The first aider should inform the person of the consequences that could apply to them for breaking local alcohol restrictions or breaking laws that apply to drinking (such as drink driving). |
| 95 | 1 | If the first aider becomes aware that the person is being aggressive because of their drinking, the first aider should suggest the person seek professional help or attend a self-help group (e.g. anger management or men's group). |
| 96 | 1 | The first aider should explain where the person can get information about problem drinking. |
| 97 | 1 | The first aider should encourage the person to see a professional to get information about problem drinking. |
| 98 | 1 | The first aider should provide some practical tips for low-risk drinking. |
| 99 | 2 | The first aider should encourage the person to read some information about Aboriginal people and problem drinking (eg. The Grog Book). |
| 100 | 2 | The first aider should encourage the person to find some information about Aboriginal people and problem drinking (eg. books, videos, local people who have stopped grog, drug and alcohol services). |
| *2.4 Encouraging the person to change* | | |
| 101 | 1 | The first aider should encourage the person to drink less. |
| 102 | 1 | f the person has expressed a desire to change their drinking behaviour, then the first aider should encourage the person to drink less. |
| 103 | 1 | If the person’s drinking is placing their physical, emotional or social health at risk, then the first aider should encourage the person to drink less. |
| 104 | 1 | The first aider should provide the person with some information about how to reduce the amount they drink. |
| 105 | 1 | The first aider should discuss ways the person could drink without it impacting negatively on others. |
| 106 | 1 | The first aider should support the person but not their drinking. |
| 107 | 1 | The first aider should refuse to support the person's drinking by not giving them money, pay their bills or buying them grog when the person wants to go out and drink. |
| 108 | 1 | The first aider should be aware that the person will not change their behaviour if they do not have to face the consequences of their actions. |
| 109 | 3 | The first aider should suggest that the person find a buddy who can help the person by sharing common goals around staying sober. |
| ***Section 3. If the person wants to change***  *3.1 Initiating change* | | |
| 110 | 1 | The first aider should be aware that abstinence from drinking may not be the person’s goal and that reducing the quantity of alcohol consumed is worthwhile. |
| 111 | 1 | If the person wants to reduce their drinking, but not stop altogether, the first aider should remain positive and encouraging towards the person. |
| 112 | 1 | The first aider should suggest that only the person can take responsibility for reducing their drinking. |
| 113 | 1 | The first aider should encourage the person to participate in social or cultural activities that do not involve drinking. |
| 114 | 1 | The first aider should support and encourage any small changes or improvements in the person's drinking behaviour. |
| 115 | 2 | If the person wishes to give up or reduce their drinking, the first aider should encourage the person to seek professional help before they change their drinking behaviour. |
| 116 | 2 | The first aider should suggest that, although changing drinking patterns is difficult, the person should not give up trying. |
| *3.2 Dealing with the social pressure to drink* | | |
| 117 | 1 | The first aider should be aware that there is often social pressure to get drunk when drinking. |
| 118 | 1 | The first aider should reassure the person that they have the right to refuse a drink when under pressure from others to drink. |
| 119 | 1 | The first aider should advise the person to be assertive when they feel pressured to drink more than they want or intend to. |
| 120 | 1 | The first aider should reassure the person that they can say “no thanks”, without explanation, when under pressure to drink. |
| 121 | 1 | The first aider should remind the person that when under pressure to drink, they can always volunteer to be the designated driver. |
| 122 | 1 | The first aider should suggest the person try to stay away from people who pressure them to drink. |
| 123 | 1 | If the community has rules about restricting access to alcohol, the first aider should encourage the person to follow them. |
| 124 | 2 | The first aider should suggest different ways the person can say no when pressured to drink such as “I don’t feel like it”, "I don't feel well" or "I am taking medication”. |
| 125 | 2 | The first aider should reassure the person that saying “no” to drinking gets easier the more they do it. |
| 126 | 2 | The first aider should suggest that, when under pressure to drink, the person can always pour themselves a soft drink and let others assume it is a mixed drink. |
| *3.3 Encouraging other supports* | | |
| 127 | 1 | The first aider should warn the person that not all family and friends will be supportive of their efforts to change their drinking behaviour. |
| 128 | 1 | The first aider should encourage the person to find ways of coping when they feel the urge to drink. For instance, the first aider should encourage the person to talk to someone they trust (such as an Elder), get back to country, participate in ceremony or art. |
| 129 | 1 | The first aider should encourage the person to find a relative, community member, friend or group that can support the person while they change their drinking behaviour. |
| 130 | 1 | The first aider should encourage the person to talk to a friend or other person they trust about their problems, not just their drinking. |
| 131 | 1 | The first aider should encourage the person to speak to people who have given up the grog. |
| 132 | 1 | The first aider should encourage the person to spend time with non-drinking family or friends. |
| 133 | 1 | The first aider should encourage the person not to push away the people who worry or complain about their drinking, as these people are a potential source of support. |
| 134 | 1 | The first aider should discuss some information about self-help programs with the person. |
| 135 | 1 | The first aider should encourage the person to use a self-help method or join a support group program, such as Alcoholics Anonymous (AA). |
| 136 | 2 | If the person has strong ties to land, the first aider should encourage the person to use these to help them stop their drinking (e.g. by going bush, going hunting or back to country). |
| ***Section 4. Seeking professional help***  *4.1 Professional help seeking* | | |
| 137 | 1 | The first aider should be aware that drinking problems need to be addressed as part of a comprehensive, holistic approach to health that includes physical, spiritual, cultural, emotional and social aspects. |
| 138 | 1 | The first aider should be aware that treatment for problem drinking should involve controlled withdrawal or “detox”, medical treatment for health problems related to drinking, as well as counselling treatments to help the person change their behaviour. |
| 139 | 1 | The first aider should be aware of the different options for seeking professional help in the person’s local area. These may include an Aboriginal health service, a drug and alcohol service, a counsellor, doctor, or help-line. |
| 140 | 1 | The first aider should advise the person to see a doctor/GP because they play an important role in helping the person care for their health while they change their drinking habits. |
| 141 | 1 | The first aider should be aware that some treatment programs aim to have an alcohol-free lifestyle, while other programs aim to reduce the person's drinking to a safer level. |
| 142 | 1 | The first aider should encourage the person to see a professional who is experienced in helping with drinking problems in the local community. |
| 143 | 1 | The first aider should help the person find a treatment program that the person feels comfortable with. |
| 144 | 1 | The first aider should encourage the person to find a professional or treatment program that specialises in treating Aboriginal people with drinking problems. |
| 145 | 2 | The first aider should be aware that drug and alcohol services that specialise in treating Aboriginal people may be hard to find, especially in rural and remote areas. |
| 146 | 2 | The first aider should encourage the person to engage professional help that will address many aspects of the person’s social and emotional wellbeing, not just their physical health. |
| *4.2 Discussing professional help with the person who wants to change* | | |
| 147 | 1 | The first aider should be aware that the person may not want professional help when it is first suggested to them. |
| 148 | 1 | The first aider should be aware that the person may find it difficult to accept professional help. |
| 149 | 1 | The first aider should be aware that it is ultimately the person’s decision to get professional help. |
| 150 | 1 | The first aider should be aware that the sooner professional help commences, the better the person’s chances for recovery. |
| 151 | 1 | The first aider should explain to the person that there are several approaches available for treating problem drinking. |
| 152 | 1 | The first aider should reassure the person that professional help is confidential. |
| 153 | 1 | The first aider should suggest to the person that it takes courage to ask for, and to accept, professional help. |
| 154 | 1 | The first aider should assure the person that the first aider will support them in getting professional help. |
| 155 | 1 | If the person is willing to seek professional help, the first aider should give them information about local options. |
| 156 | 1 | The first aider should discuss with the person what seeing a professional might involve. |
| ***Section 5. If the person does not want help***  *5.1 If the person is unwilling to change their drinking behaviour* | | |
| 157 | 1 | The first aider should explain the consequences of continuing with their current drinking behaviours. |
| 158 | 1 | The first aider should not cover up or make excuses for the person. |
| 159 | 1 | The first aider should not take on the person’s responsibilities. |
| 160 | 1 | The first aider should not join in drinking with the person. |
| 161 | 1 | The first aider should not try to control the person by bribing, nagging, threatening or crying. |
| 162 | 1 | The first aider should not feel guilty or responsible if the person is unwilling to change their drinking behaviour. |
| 163 | 1 | The first aider should be patient while waiting for the person to accept they have a problem. |
| 164 | 1 | The first aider should assure the person that the first aider will be there if the person wants help or to talk again in the future. |
| 165 | 1 | If drinking problems in the person’s community are widespread, the first aider should speak to community leaders about initiating change. |
| *5.2 If the person is unwilling to seek professional help* | | |
| 166 | 1 | The first aider should explain to the person that you don’t need to be an ‘alcoholic’ to benefit from talking to a professional. |
| 167 | 1 | The first aider should explain that the goal of professional help may be to help the person to find ways to reduce their drinking, rather than to make the person quit altogether. |
| 168 | 1 | The first aider should discuss with the person how a doctor or health worker can help them with their problem drinking by giving advice that is personal and helpful for their situation. |
| 169 | 1 | The first aider should reassure the person that seeing a doctor or health worker is private and not a shame job. |
| 170 | 1 | The first aider should not organise a group of people to confront them about their drinking. |
| 171 | 1 | The first aider should be aware that the person may only accept professional help when the consequences of their drinking become bad enough. |
| 172 | 1 | The first aider should give the person a card or phone number of a service they can use when they feel ready. |
| 173 | 1 | The first aider should be compassionate and patient while waiting for the person to accept they need it. |
| 174 | 2 | The first aider should not cover up or make excuses for the person. |
| 175 | 2 | The first aider should explain that a person with any type of problem drinking can benefit from professional help. |
| ***Section 6. Intoxication***  *6.1 What the first aider needs to know about intoxication* | | |
| 176 | 1 | The first aider should be able to recognise the signs of intoxication. |
| 177 | 1 | The first aider should be able to recognise the signs of different levels of intoxication. |
| 178 | 1 | The first aider should be aware that symptoms of other medical conditions can mimic the symptoms of intoxication. |
| 179 | 1 | The first aider should be aware that intoxication may lead to a medical emergency. |
| 180 | 1 | The first aider should be aware that, when intoxicated, the person may be at a higher risk of attempting suicide. |
| 181 | 1 | The first aider should be aware that when intoxicated the person may engage in a wide range of risky activities, such as having unprotected sex, arguments or fights, or driving a car. |
| 182 | 1 | The first aider should be aware that only time will reverse the effects of intoxication. |
| 183 | 1 | The first aider should be aware that it takes the liver about 1 hour to clear 1 standard drink from the bloodstream. Nothing will speed this up - not cold showers, sleep or coffee. |
| 184 | 1 | The first aider should be aware of what resources are available to monitor and help intoxicated people within the person’s community (eg. night patrols, sobering up shelters). |
| 185 | 1 | The first aider should be aware that if the person needs to be contained, sobering up shelters and drug and alcohol resource centres are preferable to police lock-ups, because they can help the person stay safe, learn about their drinking and its risks, and get some professional help. |
| *6.2 If the person is intoxicated* | | |
| 186 | 1 | The first aider should assess the situation for potential dangers and ensure that the intoxicated person, themselves and others are safe. |
| 187 | 1 | The first aider should monitor for signs that the person is suicidal. |
| 188 | 1 | The first aider should know how to help the affected person if they are experiencing suicidal thoughts or behaviours. |
| 189 | 1 | The first aider should keep the intoxicated person away from machines and dangerous objects. |
| 190 | 1 | The first aider should watch the intoxicated person for signs of increasing aggression. |
| 191 | 1 | If the person is heavily intoxicated and the first aider feels uncomfortable monitoring them, the first aider should take the person to sobering up centre or drug and alcohol service. |
| 192 | 2 | If the first aider has been drinking, they should enlist the help of someone who is sober to assist the person. |
| 193 | 2 | The first aider should be aware that the person may have irrational thinking. |
| *6.3 Talking to the intoxicated person* | | |
| 194 | 1 | The first aider should stay calm. |
| 195 | 1 | The first aider should talk with the intoxicated person using simple, clear language. |
| 196 | 1 | The first aider should be aware that the intoxicated person may overreact to negative words; therefore, the first aider should use positive words (such as ‘stay calm’) instead of negative words (such as ‘don’t get angry’). |
| 197 | 1 | The first aider should not laugh at, make fun of, or provoke the intoxicated person. |
| 198 | 1 | The first aider should not attempt to engage the intoxicated person in a serious conversation about their drinking behaviour. |
| 199 | 1 | The first aider should ask the intoxicated person if they have taken any medications or other drugs, in case the person’s condition deteriorates into a medical emergency. |
| *6.4 Getting the intoxicated person home or to a safe place* | | |
| 200 | 1 | The first aider should organise a safe mode of transport for the intoxicated person. |
| 201 | 1 | The first aider should DISCOURAGE the intoxicated person from driving a vehicle or riding a bike. |
| 202 | 1 | The first aider should prevent the intoxicated person from walking off alone, but only if it is safe for the first aider to do so. |
| 203 | 1 | The first aider should advise the intoxicated person not to drive the next day or engage in other tasks that involve risk of injury. |
| *6.5 What to do if the intoxicated person becomes aggressive* | | |
| 204 | 1 | The first aider's priority should be to keep themselves and others safe. |
| 205 | 1 | The first aider should de-escalate the situation as much as possible. |
| 206 | 1 | The first aider should remain as calm as possible. |
| 207 | 1 | The first aider should speak slowly and confidently. |
| 208 | 1 | The first aider should speak with a gentle, caring tone of voice. |
| 209 | 1 | The first aider should avoid arguing with the intoxicated person. |
| 210 | 1 | The first aider should not speak to the intoxicated person in a hostile or threatening manner. |
| 211 | 1 | If inside, the first aider should keep the exits clear so that the intoxicated person does not feel penned in and the first aider and others can get away easily if needed. |
| 212 | 1 | If the first aider’s attempts to de-escalate a threat of violence do not work, and there is a night patrol in the person’s community, the first aider should ask them for help. |
| 213 | 1 | If violence occurrs, the first aider should seek the appropriate emergency assistance. |
| 214 | 2 | The first aider should ensure their own safety by keeping their distance from the intoxicated person. |
| 215 | 2 | The first aider should consider taking a break from the conversation to allow the intoxicated person a chance to calm down. |
| 216 | 2 | If the person becomes aggressive, the first aider should only call the police if all other avenues of de-escalation have been exhausted. |
| ***Section 7. Withdrawal*** | | |
| 217 | 1 | The first aider should be able to recognise the symptoms of withdrawal. |
| 218 | 1 | The first aider should be aware that people who have been drinking heavily for a long time can feel very sick when they stop. |
| 219 | 1 | The first aider should be aware that some treatment programs will give medicines to help withdrawal, some will recommend a stay at a residential service to withdraw, while others can assist withdrawal within the person's home. |
| 220 | 1 | The first aider should encourage the person to get medical advice, from a doctor or drug and alcohol service, before they stop drinking. |
| 221 | 1 | If the person stops drinking heavily, and becomes delirious and confused, the first aider should get medical help. |
| 222 | 1 | If the person stops drinking heavily, and begins hallucinating, the first aider should get medical help. |
