# Supplementary material for: Development of mental health first aid guidelines for Aboriginal and Torres Strait Islander people experiencing problems with substance use: a Delphi study
Source: BMC Psychiatry. 2010 Oct 8;10:78. doi: 10.1186/1471-244X-10-78 (PMC2964528; doi:10.1186/1471-244X-10-78)
Supplement: Additional file 2 — Endorsed Statements Problem Drug Use. Endorsed first aid action statements from the problem drug use study. [file 1471-244X-10-78-S2.DOC]

Endorsed statements from the Delphi study “First Aid for Problem Drug Use”

| Statement number | Round endorsed | Endorsed statements |
| --- | --- | --- |
| ***Section 1. Problem drug use***  *1.1 What the first aider needs to know about problem drug use* | | |
| 1 | 1 | The first aider should have some general knowledge of the range of reasons why people develop problem drug use (e.g. it helps them to cope or stopping leads to unpleasant effects). |
| 2 | 1 | The first aider should be aware that there may be things happening within the family and/or the community that are contributing to the person’s drug use. |
| 3 | 1 | The first aider should be aware that the person may not be clear about why they use drugs. |
| 4 | 1 | The first aider should be aware that drugs are often used to cope with underlying emotional distress or mental illness. This is often called ‘self-medication’. |
| 5 | 1 | The first aider should be aware that mental health problems can be caused or made worse by drug use. |
| 6 | 1 | The first aider should be aware that problem drug use is not just a matter of how much of a drug the person is using, but how their use affects their life and the lives of those around them. |
| 7 | 1 | The first aider should not assume that if a person is using drugs, it means the person has a drug use problem. |
| 8 | 1 | The first aider should be aware of the short- and long- term consequences of problem drug use. |
| 9 | 1 | The first aider should be aware of the impacts of problem drug use in Aboriginal and Torres Strait Islander communities. |
| 10 | 1 | The first aider should be aware that changing problem drug use takes time. |
| 11 | 1 | The first aider should know that trying to cut back on drug use is hard, and the person will experience emotional, physical and mental stress. |
| 12 | 1 | The first aider should know the stages of change and the process it takes for somebody to change their behaviour. |
| 13 | 1 | The first aider should be aware that they cannot make the person change their problem drug use. |
| 14 | 1 | The first aider should be aware that to reduce their problem drug use, the person’s underlying emotional distress or mental health problems will usually need to be addressed. |
| 15 | 1 | The first aider should be aware of the range of treatment options and support services available to the person, such as education, counselling, therapy, rehabilitation and self-help groups. |
| 16 | 1 | The first aider should be aware that people who use drugs do so at different levels ranging from experimentation to heavy use and people need different types of help depending on their level of use. |
| 17 | 1 | The first aider should be aware that the person may make significant demands on family and members of the community to support their drug use. For example, the person may demand money or transport to obtain the drug. |
| 18 | 1 | The first aider should be aware that the person might experience frequent or permanent homelessness as a result of their problem drug use. |
| 19 | 1 | The first aider should be aware of how the drug/s the person is taking may be affecting their behaviour. |
| 20 | 1 | The first aider should know that even doing small things to help can make a difference to the person’s problem drug use. |
| 21 | 1 | The first aider should have an understanding of the different drugs used in the person's community. |
| 22 | 1 | The first aider should be aware that stigma and discrimination associated with problem drug use can be a barrier to help-seeking. |
| 23 | 2 | The first aider should be aware of the different types of help needed at each stage of change. |
| 24 | 2 | The first aider should be aware that while abstinence may be a suitable treatment aim for some people, many programs recognise that, for others, this may not be possible or realistic. |
| 25 | 2 | The first aider should be aware that they can contact a professional for information and support in relation to assisting someone with problem drug use. |
| *1.2 How to recognise problem drug use* | | |
| 26 | 1 | The first aider should be able to recognise the signs of problem drug use. |
| 27 | 1 | The first aider should be able to recognise the symptoms of drug abuse. |
| 28 | 1 | The first aider should be able to recognise the symptoms of drug dependence. |
| 29 | 1 | The first aider should respect the person’s privacy, (e.g. should not search through their things to look for signs of drug use). |
| 30 | 2 | The first aider should be aware that the signs of problem drug use may be confused with depression or stress. |
| ***Section 2. Approaching the person about their problem drug use***  *2.1 Preparing to approach the person* | | |
| 31 | 1 | The first aider should be aware that the person may react negatively when approached about their problem drug use, (e.g. the person may deny they have a problem or get angry at the first aider). |
| 32 | 1 | The first aider should be aware that it may not be easy talking to the person, as they may not consider their drug use a problem. |
| 33 | 1 | The first aider should be aware that the person may find it hard to disclose or discuss their drug use because of shame. |
| 34 | 1 | The first aider should not expect the person to tell them everything about their drug use. |
| 35 | 1 | The first aider should have some helpful contact numbers with them so the person can call for confidential help or for more information, if they are willing to receive it. |
| 36 | 1 | If the first aider does not know the person very well, the first aider should try to make a connection with the person before talking to them about their drug problem, (e.g. share some information about who they are and ask the person where they are from). |
| 37 | 2 | The first aider should be aware that they can speak with a health worker to determine how best to approach the person about their concerns. |
| *2.2 General principles for talking to the person* | | |
| 38 | 1 | The first aider should remain calm, open and honest when talking to the person about their problem drug use. |
| 39 | 1 | The first aider should not talk to the person in a confrontational way, (e.g. should not blame the person, argue or yell at them). |
| 40 | 1 | The first aider should be assertive but not aggressive. |
| 41 | 1 | The first aider should express their concerns in a non-judgmental way. |
| 42 | 1 | The first aider should listen to the person in a non-judgemental way. |
| 43 | 1 | The first aider should talk with the person in a supportive manner. |
| 44 | 1 | The first aider should use “I” statements instead of “you” statements, (e.g. “I feel worried/angry/frustrated when you…” instead of “You make me feel…”). |
| 45 | 1 | The first aider should use open questions that encourage the person to think about their drug use, (e.g. what do you think about your drug use? How do you think you can change it?). |
| 46 | 1 | The first aider should not label or call the person an ‘addict’. |
| 47 | 1 | The first aider should stick to the point (i.e. focus on the person’s drug use) and not get drawn into arguments about other issues. |
| 48 | 1 | The first aider should allow the person to talk about concerns not related to their problem drug use, (e.g. family business). |
| 49 | 1 | The first aider should not interrupt the person while they are speaking. |
| 50 | 1 | The first aider should repeat back to the person what they have said to show that they understand, (e.g. so what you are saying is…). |
| 51 | 1 | The first aider should listen carefully to the person rather than talking all the time. This allows the first aider to think carefully about what the person is saying and doing, and what the best way to help them would be. |
| 52 | 1 | The first aider should not expect the person to change their drug use right away; this conversation may be the first time they have thought of their drug use as a problem. |
| 53 | 1 | The first aider should not try to quickly fill a silence in the conversation. Silence can be used to show respect and acknowledge that the person has said something important. |
| 54 | 1 | The first aider should allow the person time to tell their story. |
| 55 | 1 | The first aider should be warm and sincere to the person, to help them feel secure about discussing their problem. |
| 56 | 1 | The first aider should talk to the person about their problem without being patronising. |
| 57 | 2 | The first aider should focus the conversation on the person’s behaviour rather than their character. |
| 58 | 2 | The first aider should discuss the issue of drug use openly with the person. This may help the person feel comfortable talking about their own drug use. |
| 59 | 2 | The first aider should tell the person that it is ok to feel the way they are feeling. |
| *2.3 When to talk to the person* | | |
| 60 | 1 | The first aider should try to organise a time to talk to the person when there will be no interruptions or distractions. |
| 61 | 1 | The first aider should try to talk with the person in a quiet, private environment |
| 62 | 1 | The first aider should try to talk with the person when both are in a calm frame of mind. |
| 63 | 1 | The first aider should be available and show a positive response when the person says they are ready to talk. |
| *2.4 What to say to the person* | | |
| 64 | 1 | The first aider should not press the person to talk if they don't want to talk. |
| 65 | 1 | The first aider should ask the person if they want help to change their problem drug use. |
| 66 | 1 | The first aider should express an offer of help and discuss with the person what the first aider is willing and able to do. |
| 67 | 1 | If the person wants help to change their problem drug use, the first aider should ask what type of help and support they would find most helpful. |
| 68 | 1 | The first aider should tell the person they will listen without judging them. |
| 69 | 1 | The first aider should provide some basic facts about problem drug use. For example, how common it is, the associated risks, what treatments are available and that people can be helped. |
| 70 | 1 | The first aider should be aware that the person may already know a lot of information about problem drug use (e.g. the risks associated with use). |
| 71 | 1 | The first aider should discuss with the person whether they have ever tried to change their drug use in the past. |
| 72 | 1 | If the person has tried to make a change, the first aider should discuss with them what was helpful and what wasn’t. |
| 73 | 1 | The first aider should offer the person some information about problem drug use. |
| 74 | 1 | The first aider should ask the person if they are aware of the risks associated with problem drug use. |
| 75 | 1 | The first aider should discuss with the person that stopping drug use is hard, it may be painful, and it takes time. |
| 76 | 1 | If the person is injecting drugs, the first aider should tell the person that it is never ok to share injecting equipment (needles, syringes, tourniquets, filters, spoons, waters) with someone else, not even if they say they are clean, if they are family, or if the person knows them really well. |
| 77 | 1 | If the person does not agree they have a problem, the first aider should let the person know that they are available to talk in the future. |
| 78 | 2 | The first aider should consider the person’s readiness to talk about their problem drug use by asking about areas of their life that it may be affecting. For example, their mood, work performance, family or community. |
| 79 | 2 | The first aider should ask whether the person considers their drug use a problem. |
| *2.5 If the person is pregnant or breastfeeding* | | |
| 80 | 1 | The first aider should discuss with the person that using drugs during pregnancy is unsafe for the baby. |
| 81 | 1 | The first aider should encourage the person to seek appropriate professional help as soon as possible. |
| 82 | 1 | The first aider should strongly encourage the person to stop using drugs while pregnant. |
| 83 | 1 | If the person has been using drugs regularly, the first aider should discuss with the person that suddenly stopping drug use without medical supervision can be dangerous for the unborn baby. |
| 84 | 1 | If the person is pregnant, and wants to stop or reduce their drug use, the first aider should tell the person that medical help is essential to do this safely. |
| 85 | 1 | The first aider should discuss with the person that using drugs while breastfeeding is unsafe for the baby. |
| 86 | 2 | The first aider should tell the person not to use drugs while breastfeeding. |
| *2.6 If the person is caring for a child* | | |
| 87 | 1 | The first aider should encourage the person not to use drugs around children. |
| ***Section 3. Information and support for the person who wants to stop using drugs***  *3.1 Self help* | | |
| 88 | 1 | The first aider should offer the person information about self-help strategies (e.g. reading books about changing drug use, or attending a support group). |
| 89 | 1 | The first aider should encourage the person to eat healthy so their body can be strong while they are trying to change their problem drug use. |
| 90 | 1 | The first aider should encourage the person to try and get a good amount of sleep so their body can work to repair itself while they are trying to change their problem drug use. |
| 91 | 1 | The first aider should encourage the person to find healthy ways to feel good instead of using drugs. |
| 92 | 1 | The first aider should encourage the person to do more of what keeps them strong (e.g. getting back to country, re-connecting with culture or talking with family). |
| 93 | 2 | The first aider should encourage the person to listen to the stories of people who have stopped using drugs. |
| *3.2 Helpful information* | | |
| 94 | 1 | The first aider should encourage the person to get a health check. |
| 95 | 1 | The first aider should help the person get some information on how to change their problem drug use. |
| 96 | 1 | The first aider should offer the person information about a range of local treatment options (e.g. self-help resources, support groups, health services or traditional healer) and allow the person to decide which would be most appropriate or useful for them. |
| 97 | 2 | The first aider should discuss with the person that using drugs to escape problems just makes the problems worse. |
| 98 | 2 | The first aider should help the person get some professional advice on how to change their problem drug use. |
| *3.3 Support* | | |
| 99 | 1 | The first aider should be positive and encouraging of any efforts the person makes to change their problem drug use. |
| 100 | 1 | The first aider should encourage the person talk to someone they trust about the efforts they are making to change their problem drug use, for example, a friend, family member, respected Elder or community support worker. |
| 101 | 1 | The first aider should encourage the person to get support from their family, friends and community while they are trying to change their drug use. |
| 102 | 1 | The first aider should always support the positive choices that the person makes (e.g. playing sport or spending time with people that don’t use drugs). |
| 103 | 1 | The first aider should not support the person’s drug use (e.g. should not give them money to buy drugs). |
| 104 | 2 | The first aider should be aware that they can speak with others who have dealt with problem drug use about effective ways to help the person. |
| *3.4 Helping the person deal with social pressure to take drugs* | | |
| 105 | 1 | The first aider should help the person to think of ways to say no when offered drugs. |
| 106 | 1 | The first aider should encourage the person to be strong and say no when they don't want to take drugs. |
| 107 | 2 | The first aider should reassure the person that saying no to drugs will get easier the more they do it. |
| *3.5 Harm reduction* | | |
| 108 | 1 | The first aider should provide the person with information about harm reduction strategies specifically for Aboriginal and Torres Strait Islander people. |
| 109 | 1 | The first aider should provide the person with information about harm reduction strategies. |
| *3.6 Laws around drug use/possession* | | |
| 110 | 1 | The first aider should have some knowledge of cultural, local, state and national laws around drug use and possession. |
| ***Section 4. When to disclose the person’s drug use*** | | |
| 111 | 1 | The first aider should not disclose the person’s problem drug use to a significant other (eg. friend or family member) unless they have the person’s consent. |
| 112 | 1 | The first aider should not disclose the person’s problem drug use to a significant other (eg. friend or family member) unless the person is a child or young person. |
| 113 | 1 | The first aider should not disclose the person’s problem drug use to a significant other (eg. friend or family member) unless the person is at risk of harm to themselves. |
| 114 | 1 | The first aider should not disclose the person’s problem drug use to a significant other (eg. friend or family member) unless the person is at risk of harming others. |
| 115 | 1 | The first aider should not disclose the person’s problem drug use to a significant other (eg. friend or family member) unless there is a child or young person being placed at risk because of the person’s problem drug use. |
| 116 | 1 | The first aider should not disclose the person’s problem drug use to a professional unless they have the person’s consent. |
| 117 | 1 | The first aider should not disclose the person’s problem drug use to a professional unless the person is a child or young person. |
| 118 | 1 | The first aider should not disclose the person’s problem drug use to a professional unless the person is at risk of harm to themselves. |
| 119 | 1 | The first aider should not disclose the person’s problem drug use to a professional unless the person is at risk of harming others. |
| 120 | 1 | The first aider should not disclose the person’s problem drug use to a professional unless there is a child or young person being placed at risk because of the person’s problem drug use. |
| 121 | 2 | The first aider should tell the person they will have to tell other people about their problem drug use if there are children at risk, or the person is at risk of harm to themselves or others. |
| ***Section 5. If the person is unwilling to change*** | | |
| 122 | 1 | The first aider should give the person a clear message that changing their drug use is the best option. |
| 123 | 1 | The first aider should tell the person they are concerned about their continuing drug use. |
| 124 | 1 | The first aider should tell the person that drug use is never completely safe. |
| 125 | 1 | The first aider should NOT deny the person basic needs. |
| 126 | 1 | The first aider should not cover up or make excuses for the person. |
| 127 | 1 | The first aider should not use drugs with the person. |
| 128 | 1 | The first aider should not provide the person with money to buy drugs. |
| 129 | 1 | The first aider should not get involved with helping the person to get drugs (e.g. driving the person to meet the dealer). |
| 130 | 1 | The first aider should be aware that if they continue to be supportive they may be able to help the person change their problem drug use in the future. |
| 131 | 1 | The first aider should tell the person what behaviour they are willing to accept from the person (e.g. the first aider won’t accept the person coming to the first aider’s home for a social visit when they are intoxicated). |
| ***Section 6. Encouraging the person to seek professional help***  *6.1 Suggesting help* | | |
| 132 | 1 | The first aider should be aware of what Aboriginal health services are available in the community. |
| 133 | 1 | The first aider should be aware of what treatment options are available in the person's local area. |
| 134 | 1 | The first aider should ask the person if they would like to get professional help. |
| 135 | 1 | The first aider should encourage the person to seek professional help. |
| 136 | 1 | The first aider should be aware that it is common for people with problem drug use to resist seeking professional help. |
| 137 | 1 | The first aider should discuss with the person why they need professional help. |
| 138 | 1 | If the person is using more than one type of drug, the first aider should strongly recommend that they seek professional help. |
| 139 | 1 | The first aider should be prepared for the person to respond negatively when professional help is suggested. |
| 140 | 1 | If the person has been using regularly, the first aider should recommend that the person seek medical advice before they attempt to stop using drugs. |
| 141 | 1 | The first aider should be aware that the person may find it difficult to accept professional help. |
| 142 | 1 | The first aider should reassure the person that professional help is confidential. |
| 143 | 1 | The first aider should give the person information about how to get professional help. |
| 144 | 1 | The first aider should tell the person that it takes courage to ask for, and to accept, professional help. |
| 145 | 1 | The first aider should not pressure the person into a single type of treatment. |
| 146 | 1 | The first aider should not take on the role of a counsellor. |
| *6.2 Types of help* | | |
| 147 | 1 | The first aider should give the person information about local options. |
| 148 | 1 | The first aider should encourage the person to talk to a health worker, family or respected Elders. |
| 149 | 1 | The first aider should tell the person that they will support them in getting professional help. |
| 150 | 2 | The first aider should help the person to find a traditional healer if the person wants to see one. |
| *6.3 Making the appointment* | | |
| 151 | 1 | The first aider should be aware that the person may not like going to health services. |
| 152 | 1 | The first aider should follow up with the person to see how the appointment went. |
| 153 | 1 | The first aider should encourage the person to ask the professional about any words they don’t understand. |
| 154 | 2 | The first aider should help the person find a professional they are happy with. |
| ***Section 7. If the person is unwilling to seek help*** | | |
| 155 | 1 | The first aider should not use negative approaches to get the person to seek professional help, such as threats, accusations, nagging or shaming. |
| 156 | 1 | The first aider should be aware that it is ultimately the person’s decision to get professional help. |
| 157 | 1 | The first aider should not feel like they have failed if the person does not seek professional help. |
| 158 | 1 | The first aider should be patient and remain positive, because opportunities may present themselves to suggest professional help again. |
| 159 | 1 | The first aider should tell the person that there is no shame in getting help to change their problem drug use. |
| 160 | 1 | The first aider should be aware that often people will only ask for help when they see that their drug use is out of control. |
| 161 | 1 | The first aider should give the person information that they can use when they are ready to seek professional help (e.g. brochures or names and locations of services). |
| 162 | 1 | The first aider should be prepared to talk to the person about professional help again in the future. |
| 163 | 1 | If the person does not want professional help, the first aider should respect their decision. |
| 164 | 2 | The first aider should continue to encourage the person to seek professional help. |
| 165 | 2 | The first aider should suggest professional help to the person again, after an episode of drug use has led to negative consequences (e.g. the person gets into trouble with family members or the law). |
| ***Section 8. Drug affected states***  *8.1 Understanding drug affected states* | | |
| 166 | 1 | The first aider should be aware that when the person is coming down from a drug, they might be miserable and sometimes paranoid. The first aider should try to maintain a safe distance and try to appear non-threatening. |
| 167 | 1 | When dealing with a group of people who have been using drugs, the first aider should take extra care for their own safety. |
| *8.2 Sniffing* | | |
| 168 | 1 | The first aider should seek medical help if the effects of the inhalants are not wearing off after the person has stopped sniffing. |
| 169 | 1 | If possible, the first aider should move the person to a safe place that is well ventilated, or open any doors and windows. |
| 170 | 1 | The first aider should stay with the person, or make sure they stay somewhere safe, until the effects of the inhalant have worn off. |
| 171 | 1 | The first aider should be aware of the risk of sudden sniffing death (i.e. the person’s heart and breathing are affected by the inhalant and sudden exercise or a shock will cause their heart to stop). |
| 172 | 1 | To reduce the risk of sudden sniffing death, the first aider should not be threatening or chase the person. |
| 173 | 1 | The first aider should tell any other people around that it is dangerous to chase or overexcite the person. |
| 174 | 1 | The first aider should try to create a calm environment for the person, (e.g. ask spectators to move). |
| 175 | 1 | The first aider should be aware that there is a high risk that inhalants may catch on fire and cause severe burns. |
| 176 | 1 | The first aider should keep the person away from anything that could cause the inhalant to catch fire, (e.g. a lit cigarette, a cigarette lighter or campfire). |
| 177 | 2 | If the person won't hand over their inhalants, the first aider should try to keep them from actively sniffing, (e.g. get the person to talk or do something with their hands so they are not sniffing). |
| *8.3 Responding to medical emergencies* | | |
| 178 | 1 | The first aider should not be afraid to tell the emergency workers what drug/s the person has been taking. The first aider and the person will not get into trouble. |
| 179 | 1 | The first aider should be aware that overdose is not a crime, and that nobody will get into trouble when the ambulance arrives. |
| 180 | 1 | The first aider should know that the police will only be called to an emergency if the ambulance officers feel they can’t control what is happening. The emergency workers first priority is to save the life of the person who is unwell. |
| *8.4 If the person becomes agitated or aggressive* | | |
| 181 | 1 | The first aider should assess the risks to themselves, the person and others, before trying to assist someone who may become aggressive. |
| 182 | 1 | The first aider should know that sometimes drug use can cause fear, anxiety, panic or paranoia, which may result in aggression. |
| 183 | 1 | The first aider should prioritise their own safety. |
| 184 | 1 | The first aider should keep a safe distance from the person. |
| 185 | 1 | The first aider should talk to the person in a calm, non-threatening manner. |
| 186 | 1 | The first aider should speak slowly and confidently. |
| 187 | 1 | The first aider should repeat things if necessary. |
| 188 | 1 | The first aider should avoid asking too many questions, as it could make the person more angry. |
| 189 | 1 | The first aider should try not to provoke the person. |
| 190 | 1 | The first aider should avoid getting into an argument with the person. |
| 191 | 1 | The first aider should avoid displaying nervous behaviour (e.g. shuffling their feet, fidgeting or making abrupt movements). |
| 192 | 1 | If inside, the first aider should try to keep the exits clear so that the person does not feel penned in and the first aider and others can get away easily if needed. |
| 193 | 1 | The first aider should try to provide the person with a quiet environment away from noise and other distractions. |
| 194 | 1 | The first aider should tell the person what they are going to do before doing it (e.g. calling for help or asking others to leave). |
| 195 | 1 | The first aider should tell the person that no-one will hurt them. |
| 196 | 1 | The first aider should reassure the person that they are there to help. |
| 197 | 1 | The first aider should continually reassess if what they are doing is helping; if not, then they should something different. |
| 198 | 2 | A non-Aboriginal first aider should be aware that Aboriginal people tend to express a higher level of emotion, but this does not necessarily indicate aggression. |
| 199 | 2 | The first aider should be aware of local resources they can call upon to help calm the person down, e.g. community members, elders or night patrol. |
| 200 | 2 | The first aider should acknowledge the person's agitation (e.g. "I can see that you are upset") |
| 201 | 2 | If the first aider is feeling unsafe, the first aider should seek other supports (e.g. involve the person’s family members or friends). |
| 202 | 2 | The first aider should ask the person what they want and then repeat what they requested. |
| 203 | 2 | The first aider should encourage the person not to use any more drugs or alcohol in the short-term. |
| *8.5 What to do if the first aider cannot de-escalate the situation* | | |
| 204 | 1 | The first aider should get help if the person is going to hurt themselves or someone else. |
| 205 | 1 | The first aider should only call the police if all other avenues of de-escalation have been exhausted and the person is at risk of harming themselves or others. |
| 206 | 2 | The first aider should remove themselves from the situation. |
