# Supplementary material for: Development of mental health first aid guidelines for Aboriginal and Torres Strait Islander people experiencing problems with substance use: a Delphi study
Source: BMC Psychiatry. 2010 Oct 8;10:78. doi: 10.1186/1471-244X-10-78 (PMC2964528; doi:10.1186/1471-244X-10-78)
Supplement: Additional file 3 — Strongly Rejected Statements. First aid action statements from both the problem drinking and problem drug use studies. [file 1471-244X-10-78-S3.DOC]

Table 1. Strongly rejected statements* from the Problem Drinking study

| First aid action statement | Questionnaire section | Panel rating^◊^ |
| --- | --- | --- |
| The first aider should deny the person basic needs, such as keeping them warm, clean and nourished. | Section 5: If the person does not want help | 77.30% |
| The first aider should hide or pour away the person’s grog. | Section 5: If the person does not want help | 72.70% |
| The first aider should enlist the help of others (such as a doctor, relative or friend) to confront the person as a group. | Section 5: If the person does not want help | 63.60% |
| The first aider should take the intoxicated person back to the first aider’s house. | Section 6. Intoxication | 59.10% |
| The first aider should not use threats unless they are prepared to follow through with them. | Section 5: If the person does not want help | 59.10% |
| If the first aider’s attempts to de-escalate a threat of violence do not work, they should NOT call the police. | Section 6: Intoxication | 50.00% |

* Strongly rejected: those rated as either ‘Unimportant’ or ‘Should not be included’ by 50% or more of the panel

^◊^ Panel rating: percentage of panel members rating a statement as ‘Unimportant’ plus percentage rating ‘Should not be included’

Table 2. Strongly rejected statements* from the Problem Drug Use study

| First aid action statement | Section | Panel rating^◊^ |
| --- | --- | --- |
| The first aider should deny the person basic needs, such as keeping them warm, clean and fed. | Section 5. If the person is unwilling to change | 90.50% |
| The first aider should NOT provide the person with information about harm reduction strategies as this might encourage drug use. | Section 3. Information and support for the person who wants to stop using drugs | 76.20% |
| The first aider should not use threats unless they are prepared to follow through with them. | Section 5. If the person is unwilling to change | 71.40% |
| The first aider should hide or throw out the person’s drugs. | Section 5. If the person is unwilling to change | 61.20% |
| The first aider should try to take the person’s drugs away, if it is safe to do so. | Section 8. Drug affected states | 52.30% |

* Strongly rejected: those rated as either ‘Unimportant’ or ‘Should not be included’ by 50% or more of the panel

^◊^ Panel rating: percentage of panel members rating a statement as ‘Unimportant’ plus percentage rating ‘Should not be included’
